# Biodegradable Polymer Ligatures and Auxiliaries in Orthodontics: An Empty Systematic Review

**DOI:** 10.1155/ijod/5936623

**Published:** 2026-09-07

**Authors:** Ramya Vijeta Jathanna, Vinod Rakesh Jathanna, Sahana Maben, Rithesh Bangera

**Affiliations:** ^1^ Department of Orthodontics and Dentofacial Orthopedics, Manipal College of Dental Sciences, Manipal Academy of Higher Education, Manipal, India, manipal.edu; ^2^ Department of Conservative Dentistry and Endodontics, Manipal College of Dental Science Mangalore, Manipal Academy of Higher Education, Manipal, India, manipal.edu; ^3^ Department of Public Health Dentistry, AB Shetty Memorial Institute of Dental Sciences (ABSMIDS), Nitte (Deemed to be University), Mangalore, India, nitte.edu.in; ^4^ Department of Orthodontics and Dentofacial Orthopedics, KVG Dental College and Hospital, Kurunjibhag, Sullia 574327, Karnataka, India, kvgdentalcollege.com

**Keywords:** biocompatible materials, biopolymers, dental materials, orthodontic

## Abstract

**Background:**

Orthodontic ligatures and auxiliaries are essential components of fixed‐appliance therapy. Conventional materials are nonbiodegradable and require periodic replacement, contributing to clinical inefficiency and environmental burden. Biodegradable polymer‐based alternatives have been proposed as potential solutions.

**Objective:**

To systematically evaluate the available evidence on biodegradable polymer ligatures and auxiliaries in orthodontics, focusing on their mechanical properties, biodegradation behavior, and clinical applicability.

**Methods:**

A systematic search was conducted in PubMed and Scopus databases from inception to January 2026. The review followed the PRISMA 2020 guidelines. Studies evaluating biodegradable polymer ligatures or orthodontic auxiliaries were included. The screening was performed independently by two reviewers.

**Results:**

A total of 149 records were identified. After removing one duplicate, 148 records were screened by title and abstract. Six studies were assessed for full‐text eligibility; however, none of them met the inclusion criteria. The excluded full‐text studies primarily involved conventional elastomeric ligatures, nonbiodegradable polymers, and materials lacking biodegradation properties.

**Conclusion:**

No studies have evaluated biodegradable polymer ligatures or auxiliaries in orthodontics. This highlights a significant research gap and underscores the need for the development and investigation of biodegradable orthodontic biomaterials.

## 1. Introduction

Orthodontic treatment relies heavily on ligatures and auxiliaries to secure archwires and facilitate controlled tooth movement. Conventional ligatures are typically composed of elastomeric polymers or metallic materials, such as stainless steel. Although clinically effective, these materials are nonbiodegradable and require frequent replacement due to forced decay and material degradation, leading to increased chairside time and material waste [1–3].

Although orthodontic ligatures constitute relatively small biomaterials, they are routinely replaced throughout the treatment and represent one of the most frequently discarded disposable components in fixed orthodontic therapy. Simultaneously, advances in biodegradable polymers have transformed numerous medical disciplines; however, their application in orthodontic ligation systems remains unexplored. Therefore, establishing whether any evidence exists is an important first step before future material development, making an empty systematic review both timely and scientifically justified.

Biodegradable polymers, such as polylactic acid (PLA), polycaprolactone (PCL), and polylactic‐co‐glycolic acid (PLGA), have gained prominence in biomedical applications owing to their favorable biocompatibility, controlled degradation, and resorption characteristics [4]. These materials have been successfully utilized in drug delivery systems, tissue engineering scaffolds, and resorbable fixation devices [5–9]. However, their translation into orthodontic ligatures presents unique challenges. Unlike resorbable fixation devices or tissue scaffolds, orthodontic ligatures must maintain predictable mechanical properties throughout a defined period of clinical service while functioning in complex oral environments. Therefore, biodegradability should not compromise clinical performance. The application of biodegradable polymers in orthodontic ligatures and auxiliaries could potentially eliminate the need for removal, reduce the clinical burden, and improve environmental sustainability. Rather than advocating immediate clinical implementation, the present review aimed to determine whether scientific evidence currently exists regarding biodegradable polymer ligatures and auxiliaries and to identify knowledge gaps that may guide future biomaterials research.

## 2. Materials and Methods

### 2.1. Protocol and Registration

This systematic review was conducted in accordance with the PRISMA 2020 guidelines and prospectively registered in PROSPERO (CRD420261345406) prior to the commencement of the study screening [10].

### 2.2. Eligibility Criteria

The inclusion and exclusion criteria are described in Table 1.

**Table 1:** Inclusion and exclusion criteria.

Inclusion criteria	Exclusion criteria
Studies evaluating biodegradable polymer ligatures or orthodontic auxiliaries	Studies evaluating conventional nonbiodegradable elastomeric ligatures or auxiliaries only
Studies involving orthodontic treatment or orthodontic appliances	Nonorthodontic biomedical applications
Studies evaluating polymer‐based biodegradable materials (PLA, PCL, PGA, PLGA, bio‐polymer, resorbable polymer)	Study on brackets/wires/ implants without auxiliaries
Studies reporting mechanical, biological, degradation, or clinical outcomes	Bone graft/scaffold/membrane not related to auxiliaries
In vitro human studies	Animal studies
Experimental laboratory or clinical studies	Review article/systematic review/meta‐analysis, case report/case series (<5 samples) editorial/letter/conference abstract only, expert opinions
Articles published in English	Non‐English article
Full‐text articles obtainable through institutional subscriptions, open‐access platforms, publisher websites, or other legitimate academic access methods	Studies for which the complete full text could not be obtained despite institutional or publisher access
Peer‐reviewed journal articles	Nondental/nonorthodontic application

### 2.3. Information Sources and Search Strategy

A comprehensive electronic search was conducted in PubMed and Scopus, which together provide broad coverage of biomedical, dental, and multidisciplinary scientific literature. on February 1, 2026. ClinicalTrials.gov was also searched to identify unpublished or ongoing studies. The reference lists of all potentially eligible studies were manually screened to identify additional publications. Although Embase, Web of Science, and the Cochrane Library were not searched, PubMed and Scopus were selected because they encompass most of the peer‐reviewed orthodontic and biomaterials literature. This limitation was acknowledged when interpreting the findings.

Keywords included combinations of “biodegradable polymer,” “orthodontic ligature,” “orthodontic auxiliary,” and “resorbable orthodontic material.” Unpublished literature was searched in https://clinicalTrials.gov (https://www.clinicaltrials.gov) using the search terms “orthodontics” AND “ligature.” Hand‐searching of the reference lists of the retrieved full‐text articles was also conducted. The complete database‐specific electronic search strategies, including Boolean operators and database‐specific syntax, are as follows:

Pubmed: (("Orthodontics"[Mesh] OR orthodontic ^∗^ OR orthodontics) AND (ligature ^∗^ OR auxiliary OR auxiliaries OR elastic ^∗^ OR elastomeric ^∗^) AND (biodegradable OR bioresorbable OR resorbable OR biopolymer ^∗^ OR “polylactic acid” OR PLA OR PCL OR PLGA OR PGA)).

Scopus: (TITLE‐ABS‐KEY (orthodontic ^∗^ OR orthodontics OR dental) AND TITLE‐ABS‐KEY (ligature ^∗^ OR auxiliary OR auxiliaries OR elastomeric ^∗^ OR elastic ^∗^) AND TITLE‐ABS‐KEY (biodegradable OR bioresorbable OR resorbable OR biopolymer ^∗^ OR PLA OR PCL OR PLGA OR PGA)).

### 2.4. Study Selection

All records were imported into Rayyan software. After duplicate removal, the titles and abstracts were screened independently by two reviewers. The full texts of potentially eligible studies were obtained through institutional subscriptions, open‐access platforms, publisher websites, or other legitimate academic access methods.

### 2.5. Data Extraction and Risk of Bias

Data extraction and risk of bias assessment were planned but were not performed because of the absence of eligible studies.

## 3. Results

### 3.1. Study Selection

**Figure 1:**
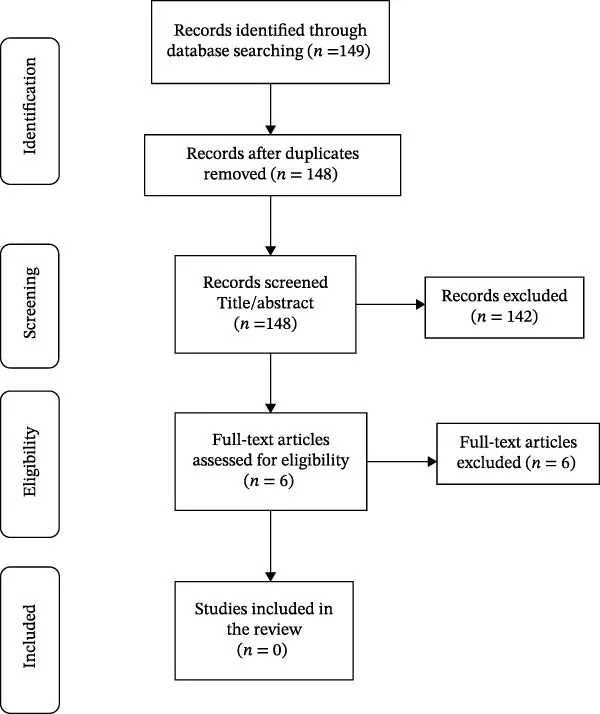
PRISMA flowchart.

A total of 149 records were identified (PubMed: 53; Scopus: 87; and https://clinicalTrials.gov: 9). After the removal of one duplicate, 148 records were screened based on their titles and abstracts. Six studies were selected for full‐text assessment. However, none of them met the inclusion criteria. Therefore, no studies were included in the qualitative or quantitative syntheses. The PRISMA flowchart 2020 illustrates the initial search for screening articles and full article reading, as shown in Figure 1.

### 3.2. Reasons for Exclusion (Full‐Text Stage)

The details of the exclusion of the six full‐text articles are provided in Table 2.

**Table 2:** Summary of excluded full‐text studies with reasons (*n* = 6).

Serial number	Study (first author, year)	Material/intervention	Primary focus	Reason for exclusion
1	McDonald et al., 2024 [11]	Elastomeric chains with SNAP + Elastollan coating	Nitric oxide release and antimicrobial activity	Not a biodegradable polymer; drug‐delivery modification without resorption or degradation evaluation
2	Bian et al., 2024 [12]	Elastomeric ligatures modified with DMAHDM	Antibacterial activity and biofilm control	Conventional elastomeric ligature with antibacterial modification; no biodegradable or resorbable component
3	Mehrbakhsh et al., 2024 [13]	Thermoplastic polyurethane ligatures	Polymer synthesis and mechanical properties	No biodegradation or resorption properties assessed
4	Bian et al., 2022 [2]	Conventional elastomeric ligatures	Surface degradation and tensile strength over time	Aging study of nonbiodegradable material; no controlled biodegradation
5	Ebrahiminik et al., 2021 [14]	Elastomeric ligatures exposed to PVP‐I, CHX, H_2_O_2_	Mechanical property changes	Conventional material; no biodegradable polymer or degradation outcome
6	Balhoff et al., 2011 [15]	Commercial elastomeric chains	Force decay and mechanical performance	Mechanical study of nonbiodegradable elastomers; no resorbable material

## 4. Discussion

This systematic review found no published in vitro, in vivo, or clinical studies evaluating biodegradable polymer‐based ligatures or auxiliaries in orthodontics, underscoring a substantial and previously underreported gap in the literature on orthodontic biomaterials. Despite a comprehensive search strategy across major biomedical databases and adherence to the PRISMA 2020 guidelines, no eligible studies were identified; consequently, no qualitative or quantitative synthesis could be performed. The absence of evidence constitutes an important finding, highlighting the unmet need for innovation and scientific inquiry in this domain.

Over the past two decades, biodegradable polymers such as PLA, PCL, and PLGA have been extensively researched and successfully implemented in several medical and dental applications, including resorbable fixation devices, guided tissue regeneration membranes, sutures, and drug delivery systems [16, 17]. These materials are valued for their biocompatibility, predictable degradation profiles, and capacity to be engineered for specific mechanical and biological requirements [18, 19]. However, the present review demonstrates that such translational progress has not been extended to orthodontic ligatures or auxiliaries despite their routine, high‐volume use in clinical practice.

Several factors may account for this gap in translation. First, conventional elastomeric ligatures are inexpensive, easy to manufacture, and widely accepted by clinicians, creating limited commercial incentives for the development of alternative materials. Second, orthodontic ligatures are subjected to a uniquely challenging oral environment characterized by cyclic mechanical loading, moisture, temperature fluctuations, enzymatic activity, and microbial biofilms [20]. Designing a biodegradable material that can maintain sufficient force retention and structural integrity over a clinically relevant time while undergoing controlled degradation is a significant technical challenge. Orthodontic ligatures differ fundamentally from many biodegradable medical devices because they must preserve tensile strength, elasticity, and force delivery throughout a clinically defined service interval. Premature degradation can compromise ligation efficiency and treatment mechanics, whereas excessively delayed degradation can negate the intended clinical and environmental benefits. During the screening process, multiple studies were identified that explored modified elastomeric ligatures, including those incorporating antibacterial agents, nitric oxide donors, drug‐releasing components, or surface modifications to improve mechanical performance. While these investigations reflect a growing interest in enhancing the functionality of orthodontic ligatures, the materials evaluated remained fundamentally nonbiodegradable and therefore failed to meet the inclusion criteria for this review. This distinction is important because the surface degradation, aging, or force decay of conventional elastomers should not be conflated with true biodegradation or bioresorption.

The intentionally narrow eligibility criteria ensured that only studies directly evaluating biodegradable orthodontic ligatures and auxiliaries were included. Consequently, investigations involving animal models, tissue engineering scaffolds, bone fixation devices, or biodegradable polymers developed for nonorthodontic applications were excluded because they could not directly answer the review question. Nevertheless, these studies may provide valuable foundational information regarding polymer chemistry, degradation behavior, biocompatibility, and manufacturing approaches that could facilitate the future development of biodegradable orthodontic auxiliaries.

The findings of this review highlight several priority areas for future research. These include the development of novel biodegradable polymer formulations specifically tailored for orthodontic ligation, optimization of mechanical properties such as tensile strength, elasticity, and force decay, and systematic evaluation of degradation kinetics under simulated oral conditions. In addition, comprehensive biocompatibility testing and well‐designed clinical studies are essential to assess the safety, clinical performance, patient acceptance, and cost‐effectiveness before such materials can be translated into routine orthodontic practice.

In the context of the increasing emphasis on environmental sustainability and the reduction of biomedical plastic waste, the absence of research on biodegradable orthodontic ligatures is particularly notable. Orthodontic treatment often spans several months to years, during which ligatures and auxiliaries are repeatedly replaced, cumulatively contributing to clinical waste [21]. If successfully developed, biodegradable alternatives could offer both clinical and environmental advantages.

Although environmental sustainability has become an increasingly important consideration in healthcare, the quantity of elastomeric material generated per orthodontic patient is relatively small. Consequently, the environmental advantages of biodegradable ligatures should not be overstated and must be balanced against the considerable engineering, manufacturing, regulatory, and clinical challenges associated with the development of materials capable of maintaining predictable mechanical performance during treatment.

The oral environment presents significant challenges for the controlled biodegradation of materials. Variations in salivary pH, ionic composition, enzymatic activity, microbial biofilms, dietary habits, temperature fluctuations, and patient‐specific biological variability may influence degradation kinetics. Designing biodegradable polymers capable of maintaining consistent mechanical performance despite these variables remains a substantial challenge in materials engineering. In addition to mechanical performance, the biological safety of the degradation products requires careful consideration. Any degradation products released intraorally may come into contact with the oral tissues or be inadvertently swallowed. Therefore, comprehensive investigations of cytotoxicity, local tissue compatibility, inflammatory response, degradation pathways, and long‐term toxicological safety are essential before biodegradable orthodontic auxiliaries can be considered for clinical use. A more feasible approach may involve developing materials that remain mechanically stable throughout their intended clinical service life, exhibiting environmentally responsive degradation only after disposal. This approach preserves predictable orthodontic performance while potentially reducing long‐term environmental persistence. Nevertheless, additional challenges related to the management of biologically contaminated clinical waste require careful consideration.

Overall, this systematic review established a clear baseline by demonstrating that no evidence currently exists to support or refute the use of biodegradable polymer ligatures or auxiliaries in orthodontics. By explicitly identifying this knowledge gap, the present study provides a strong rationale and direction for future interdisciplinary research at the intersection of orthodontics and biomaterials.

Finally, although no eligible published studies were identified, the possibility that relevant laboratory investigations, industrial research, patent‐related developments, or proprietary data remain unpublished cannot be ruled out. Consequently, the present review reflects the currently accessible scientific literature rather than the complete spectrum of ongoing material developments.

## 5. Limitations

The principal limitation of this systematic review was the absence of eligible in vitro, in vivo, or clinical studies, which prevented the evaluation of both the clinical performance and material characteristics of biodegradable polymer ligatures or auxiliaries in orthodontics. Consequently, no conclusions can be drawn regarding their mechanical reliability, degradation behavior, biological safety, or suitability for routine clinical use. However, this limitation also represents the key finding of this review as it demonstrates that biodegradable ligation systems have not yet been explored at any experimental or clinical level.

From a clinical perspective, the lack of evidence precludes the assessment of essential practical parameters, including ligation efficiency, force control over activation intervals, handling characteristics, durability under oral conditions, and patient comfort. Outcomes of direct relevance to orthodontic practice, such as failure rates, replacement frequency, chairside efficiency, and impact on treatment predictability, remain unexplored. The absence of clinical or preclinical studies highlights the uncertainty surrounding the feasibility of biodegradable ligatures in managing continuous mechanical demands within the oral environment.

From a materials science standpoint, the lack of laboratory investigations limits the understanding of how biodegradable polymers would perform when engineered at the ligature scale and subjected to cyclic tensile loading, moisture exposure, temperature variation, and enzymatic activity. Fundamental properties, such as tensile strength retention, elastic recovery, creep resistance, and the interaction between degradation kinetics and mechanical performance, have not been characterized. Consequently, no structure–property relationships can be inferred to guide rational material design or optimization.

Although several excluded studies evaluated modified conventional elastomeric ligatures incorporating antibacterial agents, drug‐releasing components, or surface treatments, these materials remained fundamentally nonbiodegradable. Their exclusion was methodologically appropriate because surface aging or functional modification of elastomers does not constitute controlled biodegradation or bioresorption. Nevertheless, this pattern underscores a broader limitation within the existing research landscape, where the incremental modification of established materials has been prioritized over the development of fundamentally new biodegradable orthodontic ligation systems.

The electronic search was restricted to PubMed, Scopus, and https://ClinicalTrials.gov databases. Therefore, relevant studies indexed exclusively in other databases may have been missed.

### 5.1. Significance of an Empty Systematic Review

Empty systematic reviews are increasingly recognized as valuable scientific contributions because they identify genuine evidence gaps rather than demonstrating ineffective interventions. The absence of eligible studies should not be interpreted as evidence that biodegradable orthodontic ligatures are ineffective. This indicates that this area has not yet progressed to experimental investigation. By explicitly documenting this absence of evidence, the present review establishes a research baseline, identifies priorities for biomaterial development, and may help avoid duplication of future literature searches while encouraging interdisciplinary collaboration between orthodontists, materials scientists, polymer engineers, and biomedical researchers.

## 6. Conclusion

Currently, there is no evidence supporting the use of biodegradable polymer ligatures or auxiliaries in orthodontics. This empty systematic review identifies a significant knowledge gap but does not imply that biodegradable ligatures are presently suitable for clinical application. Future investigations should establish the feasibility of developing materials capable of maintaining predictable mechanical performance during orthodontic treatment while demonstrating controlled degradation, acceptable biocompatibility, and environmental sustainability. Such multidisciplinary research is essential before biodegradable orthodontic auxiliaries can be considered for routine clinical use.

## Author Contributions

**Ramya Vijeta Jathanna**: conceptualization, methodology, investigation, data curation, formal analysis, writing – original draft, project administration. **Vinod Rakesh Jathanna**: methodology, validation, supervision, writing – review and editing. **Sahana Maben**: investigation, data curation, validation, writing – review and editing. **Rithesh Bangera**: investigation, validation, writing – review and editing.

## Funding

This study did not receive any specific funding.

## Disclosure

All authors have read and approved the final version of the manuscript. Ramya Vijeta Jathanna had full access to all of the data in this study and takes complete responsibility for the integrity of the data and the accuracy of the data analysis.

## Ethics Statement

Ethical approval was not required for this systematic review because it involved the analysis of published literature only and did not involve human participants, animals, or identifiable patient data.

## Conflicts of Interest

The authors declare no conflicts of interest.

## Data Availability

Data sharing is not applicable to this article, as no new data were created or analyzed in this study.
